# 176. Topical Antibiotic and Antiseptic Use in the Operating Room: An Opportunity for Antimicrobial Stewardship?

**DOI:** 10.1093/ofid/ofab466.378

**Published:** 2021-12-04

**Authors:** Josef Hadib Nissan, Nina Naeger Murphy, Nilam Patel, Mary Borovicka, Michelle Hecker, David Gothard

**Affiliations:** 1 The MetroHealth System, Cleveland, Ohio; 2 MetroHealth Medical Center, Cleveland, Ohio; 3 Metro Health Medical Center, Cleveland, OH

## Abstract

**Background:**

Data suggest that topical antibiotic and antiseptic use in the operating room is common but not commonly monitored by antimicrobial stewardship programs. Although some data suggest a benefit in certain surgical procedures, the CDC and WHO advise against the routine use of topical antibiotics in surgery due to uncertainty and heterogeneity in the overall data.

**Methods:**

We conducted a retrospective 28-day period prevalence study of topical antibiotic and antiseptic use during surgical procedures performed in the operating room by 6 surgical specialties at a tertiary care medical center. For the subset of patients undergoing orthopedic surgeries, we evaluated the types of topical antibiotics received and the rates of surgical site infections (SSI) and adverse drug events within 28 days of the procedure.

**Results:**

Of 744 surgical procedures reviewed, topical antibiotics were used in 127 (17.1%), topical antiseptics in 71 (9.5%), and both in 18 (2.4%) (Table 1). Antiseptic use was higher in orthopedics relative to all other surgical specialties while topical antibiotic use was higher in neurosurgery. Hand, vascular and plastics had distinguishably lower use. In the orthopedic subgroup, after exclusions, 218 procedures were evaluated. Topical antibiotics were used in 42 (19.2%). Topical antibiotic therapy was more likely to be administered if prosthetic material was implanted, the procedure was emergent, or if a *Staphylococcus aureus* infection was present. Vancomycin was the most commonly used topical antibiotic and powder was the most commonly used type of application. As shown in table 2, SSI occurred more often when both topical antibiotics and antiseptics were applied; however, SSI events were relatively uncommon, and these were more likely to have infection present at the time of surgery. Adverse events were rare.

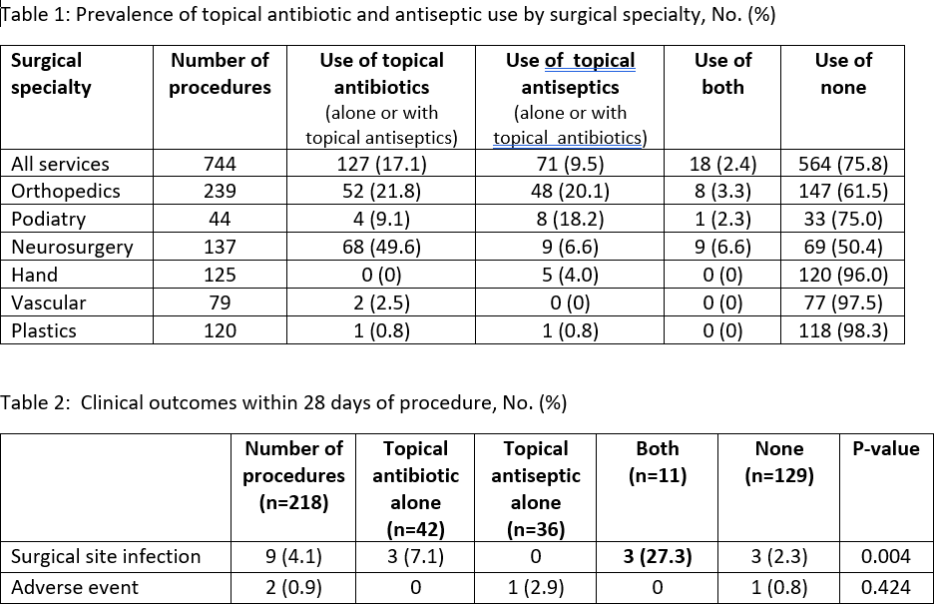

**Conclusion:**

In our institution we noted significant variability in use of topical antibiotic and antiseptic therapy among surgical specialties as well as within the orthopedic surgical specialty. Although opportunities to standardize use/nonuse of these therapies exist, this may be challenging due to the uncertainty and heterogeneity of currently available data.

**Disclosures:**

**All Authors**: No reported disclosures

